# Prevalence and correlates of suicidal behaviour among adults in Malawi: a nationally representative cross-sectional survey in 2017

**DOI:** 10.1186/s13033-021-00483-x

**Published:** 2021-06-06

**Authors:** Supa Pengpid, Karl Peltzer

**Affiliations:** 1 ASEAN Institute for Health Development, Mahidol University, Salaya, Phutthamonthon, Nakhon Pathom, Thailand; 2 Department of Research Administration and Development, University of Limpopo, Turfloop, South Africa; 3 Department of Psychology, University of the Free State, Bloemfontein, South Africa; 4 Department of Psychology, College of Medical and Health Science, Asia University, Taichung, Taiwan

**Keywords:** Suicide attempt, Suicidal behaviour, Risk factors, Adults, Malawi

## Abstract

**Background:**

The study aimed to assess the prevalence and associated factors of suicidal behaviour (SB) among adults in Malawi.

**Methods:**

Data were analysed from 4187 persons aged 18–69 years (median age: 32 years) that took part in the nationally representative cross-sectional “2017 Malawi STEPS survey.”

**Results:**

The prevalence of ever suicide attempt was 0.8%, and the prevalence of SB (ideation, plan and/or attempt) in the past year was 7.9% (6.0% among men and 9.6% among women). In adjusted logistic regression analysis, among men, being 30 years and older and having an alcohol family problem were positively and obesity was negatively associated with SB in the past year. Moreover, in univariate analysis, early smoking initiation was positively and not using active transportation was negatively associated with SB in the past year. Among women, having an alcohol family problem, death from suicide of a close family member, having a cardiovascular incident (heart attack, stroke, or angina) and not using active transportation increased the odds of SB in the past year. In addition, in univariate analysis, low systolic blood pressure (< 100 mmHg), not taking antihypertensive medication, and current alcohol use were associated with past year SB.

**Conclusion:**

Almost one in ten participants engaged in SB in the past year and several associated factors were identified that can inform intervention programmes.

**Supplementary Information:**

The online version contains supplementary material available at 10.1186/s13033-021-00483-x.

## Background

“Every year, almost 800,000 persons die from suicide, and four of five of them are from low- and middle-income countries” [1]. “For every suicide, there are many more people who attempt suicide every year.” [1] To assess and monitor the national prevalence of suicidal behaviour (SB) is needed to guide suicide prevention strategies [2]. There are scanty national data on the epidemiology of SB, especially in low resourced countries in Africa [2]. To fill this gap, this study provides an epidemiological national profile on SB among adults in Malawi, a low-income landlocked country in Southern Africa. The age-standardized suicide rate, 10.8 per 100,000 in low-income countries and in Malawi 7.8 per 100,000 (3.2 among women and 13.7 among men) [3]. Based on six sampled districts in Malawi in 2017, “9 out of 100,000 people in Malawi commit suicide annually.” [4] Malawi’s total population is 21.2 million, 17.4% live in urban areas, 80% are employed in agriculture, life expectancy at birth was 63.2 years, 62.1% can read and write and most (77.3%) are Christians by religion [5]. It has an HIV/AIDS adult prevalence rate of 9.2% and one million are living with HIV in Malawi [5].

“World Mental Health Survey” data showed a lifetime prevalence of 2.9% suicide attempt in South Africa [2] and 0.7% in Nigeria [6], and the prevalence of SB in the past year was 2.1% suicidal ideation, 0.7% suicide plans, and 0.4% suicide attempts in ten low- and middle-income countries [7]. In a community study in Ethiopia, the prevalence of SB in the ypas year was 7.0% ideation, 4.6 plans, and 3.7% attempts, and among those with a suicide attempt 26.0% sought treatment [8]. Among population-based adult samples, 51.9–60.9% of those with suicidal thoughts (suicide ideation or plan) in high-income countries received treatment, 18.5–30.9% in middle-income countries and 13.6–17.8% in low-income countries received treatment, while for those with suicide attempts, 64.7–70.3% in high income countries received treatment, 47.9–49.1% in middle-income countries, and 14.8–48.1% in low-income countries received treatment [9].

Factors associated with SB among adults may include sociodemographic factors, having psychosocial distress, physical factors, and health risk behaviours. Sociodemographic factors associated with SB may include family history of suicide, younger age, female sex [7, 8, 10], and lower socioeconomic status [11–13]. Psychosocial distress risk factors of SB may include mental illness, including major depression and alcohol use disorders [1], experiencing adverse life events [1, 14, 15], and unemployment [11–13]. Physical factors associated with SB may include suffering from physical chronic conditions, such as cardiovascular disease, epilepsy, and diabetes [11–13, 16, 17], hypercholesrolemia [16, 18] and low blood pressure [19], and health risk behaviours associated with SB, which may include high sedentary behaviour and physical inactivity [20–22], substance use disorders [23–25], passive smoking [26, 27], early substance use [28, 29] and overweight or obesity [30, 31].

It has been hypothesised that psychosocial distress, physical factors, health risk behaviours, and sociodemographic factors are associated with SB in Malawi. The study aimed to assess for the first time the national prevalence and associated factors of SB among persons aged 18–69 years in Malawi.

## Methods

Data were analysed from adults that participated in the cross-sectional nationally representative “2017 Malawi STEPS survey” [32]. Based on a multistage cluster sample design, the “2017 Malawi STEPS Survey” produced nationally representative population-based data for persons 18–69 years in Malawi [32]. The data and details of the study methodology can be publicly accessed [32].

### Measures

*Outcome variables* included lifetime suicide attempt, SB in the past year (ideation, plan and attempt), methods used for suicide attempts, and care seeking for SB (questionnaire items are shown in Additional file 1) [32].

*Sociodemographic* variables included highest formal education, age, employment and marital status, and sex.

*Psychosocial distress* indicators were passive smoking, history of attempted suicide or death from suicide of a close family member, and alcohol family problems (details in Additional file 1) [32].

*Physical factors and health risk behaviour* information consisted of measured blood pressure; those who were diagnosed with hypertension were asked if they were taking antihypertensive medication in the past 2 weeks; Body Mass Index (measured “ < 18.5 kg/m^2^ underweight, 18.5–24.4 kg/m^2^ normal weight, 25–29.9 kg/m^2^ overweight and ≥ 30 kg/m^2^ obesity”), measured diabetes (“fasting plasma glucose levels ≥ 7.0 mmol/L; or using insulin or oral hypoglycaemic drugs”); raised total cholesterol (TC) (“fasting TC ≥ 5.0 mmol/L or currently on medication for raised cholesterol”); history of “heart attack or chest pain from heart disease (angina) or a stroke (cerebrovascular accident or incident)”, age of smoking initiation, current smokeless tobacco consumption, current alcohol use, alcohol dependence, past month passive smoking, and based on the “Global Physical Activity Questionnaire” no active transportation (“walking or using a bicycle for at least 10 min to get to and from places”) and sedentary behaviour (≥ 7 h/day) [32]. *Alcohol dependence* was sourced from three items the “Alcohol Use Disorder Identification Test = AUDIT” (items 4–6), e.g., “How often during the last year have you found that you were not able to stop drinking once you had started?” Responses ranged from “0 = never to 4 = daily or almost daily”. Scores were summed, with ≥ 4 scores indicating alcohol dependence [33].

### Data analysis

Statistical analyses were done with “STATA software version 15.0 (Stata Corporation, College Station, Texas, USA),” taking into account the complex study design. Unadjusted and adjusted logistic regression was utilised to estimate predictors of ever suicide attempt and SB (ideation, plan and/or attempt) in the past year. Variables found significant in univariate analyses were subsequently added in the multivariable regression model. Covariates were included based on literature review [1, 10–31]. The missing values were not included in the analysis. P < 0.05 was accepted as significant.

## Results

### Sample characteristics

The sample consisted of 4,187 individuals (median age: 32 years, 18 years interquartile range, age range: 18–69 years), 51.7% were female, 58.7% had Standard 5 or more education, 73.0% were married or cohabiting, 43.8% were employed or students, 89.1% lived in rural areas and 89.1% were Chewa speakers. Almost one in ten participants (8.3%) reported alcohol family problems, 5.4% and 2.5% had a close family member who attempted suicide and died from suicide, respectively, and 20.7% were exposed to passive smoking. Almost one in five participants (18.5%) were overweight/obese, 6.4% had a systolic blood pressure of < 100 mmHg, 5.6% were not taking antihypertensive medications, 1.7% had prediabetes and 1.3% had diabetes, 8.2% had raised total cholesterol, 6.5% had a history of angina, heart attack, or stroke, 0.5% initiated smoking early (≤ 14 years), 1.4% were current smokeless tobacco users, 5.0% had alcohol dependence, 90.4% had inadequate intake of fruit and vegetables, 8.0% did not engage in active transportation and 5.6% engaged in sedentary behaviour (see Table 1).

**Table 1:** Sample and suicidal behaviour characteristics among 1486 men and 2702 women aged 18–69 years in Malawi

Variable	Total sample	Suicidal behaviour^a^ (past 12 months)	
		Men	Women
	N (%)	N (%)	N (%)
Socio-demographics			
All	4187	92 (6.0)	239 (9.6)
Age (years)			
18–29	1371 (45.5)	16 (2.7)	85 (8.9)
30–49	1890 (39.7)	55 (9.6)	106 (10.3)
50–69	926 (14.7)	21 (6.6)	48 (10.0)
Education			
Secondary or more	1057 (24.7)	29 (6.4)	44 (7.8)
Standard 5–8	1227 (34.0)	34 (5.4)	77 (12.1)
Standard 1–4	1318 (31.3)	27 (5.6)	89 (10.0)
None	581 (10.0)	2 (9.8)	28 (5.5)
Marital status			
Never married	418 (14.4)	11 (5.4)	11 (6.0)
Married/cohabiting	2888 (73.0)	70 (6.4)	152 (9.7)
Separated/divorced/widowed	866 (12.6)	11 (3.2)	76 (11.2)
Employment status			
Nonpaid or unemployed	2329 (56.2)	29 (5.3)	167 (10.5)
Employed or student	1756 (43.8)	62 (6.5)	70 (7.7)
Residence			
Rural	3343 (89.1)	82 (6.1)	188 (9.5)
Urban	844 (10.9)	10 (5.2)	51 (10.2)
Language of interview			
Tumbuka	145 (4.3)	3 (6.2)	4 (5.4)
Chewa	3535 (78.3)	62 (5.5)	193 (9.2)
English	506 (17.5)	27 (8.0)	42 (12.7)
Psychosocial distress			
Alcohol family problem	294 (8.3)	27 (19.1)	27 (23.0)
Family member attempted suicide	172 (5.4)	9 (6.0)	20 (22.8)
Family member died from suicide	96 (2.5)	4 (3.2)	13 (27.6)
Passive smoking	700 (20.7)	23 (8.7)	44 (12.2)
Physical factors and risk behaviours			
Systolic blood pressure (< 100 mmHg)	289 (6.4)	7 (14.8)	19 (8.9)
Not taking anti-hypertension medication	301 (5.6)	2 (2.4)	34 (15.3)
Body mass index			
Normal	2756 (73.0)	65 (5.9)	151 (10.2)
Underweight	277 (8.5)	11 (4.1)	16 (18.1)
Overweight	655 (13.5)	12 (6.9)	34 (7.6)
Obesity	319 (5.0)	3 (1.4)	22 97.1)
Diabetes status			
No	3590 (97.0)	77 (5.6)	203 (9.1)
Prediabetes	103 (1.7)	2 (6.7)	9 (19.7)
Diabetes	73 (1.3)	3 (11.5)	5 (11.2)
Raised total cholesterol	457 (8.2)	9 (10.0)	27 (6.0)
Heart attack, angina or stroke	312 (6.5)	7 (5.5)	38 (18.6)
Early smoking initiation (≤ 14 years)	26 (0.5)	4 (34.4)	1 (17.6)
Current smokeless tobacco use	78 (1.4)	1 (1.1)	9 (16.7)
Current alcohol use	558 (17.3)	34 (7.6)	11 (19.4)
Alcohol dependence	152 (5.0)	10 (9.5)	2 (7.7)
No active transport	365 (8.0)	5 (2.0)	44 (19.6)
Sedentary behaviour	233 (5.6)	10 (12.7)	16 (10.6)

^a^Suicidal ideation and/or suicide plan and/or suicide attempt

### Suicidal behaviour characteristics

The prevalence of ever suicide attempt was 0.8%, and the prevalence of past year SB (ideation 7.2%, plan 3.9%, and attempt 0.4%) was 7.9% (6.0% among men and 9.6% among women, *P* < 0.001) (see Table 1). Major suicide attempt methods were to “hang on a rope” (33.3%), “overdose of medication (e.g., prescribed, over-the-counter)” (21.7%) and “poisoning with pesticides (e.g., rat poison, insecticide, weed-killer)” (17.2%). Only a few participants (4.1%) got medical treatment following the last suicide attempt, of which none was admitted to hospital. Almost one in three participants with suicidal ideation (29.5%) had sought professional care.

### Associations with SB in the past year

In adjusted logistic regression analysis, among men, being 30 years and older and having an alcohol family problem were positively and obesity was negatively associated with SB in the past year. Moreover, in univariate, early smoking initiation was positively and not using active transportation was negatively associated with SB in the past year.

Among women, having an alcohol family problem, death from suicide of a close family member, having a cardiovascular incident (heart attack, stroke, or angina), and not using active transportation were associated with SB in the past year. Moreover, in univariate analysis, low systolic blood pressure (< 100 mmHg), not taking antihypertensive medication, and current alcohol use were associated with SB in the past year (see Table 2).

**Table 2:** Associations with suicidal behaviour (ideation, plan and/or attempt) in the past year

Variable	Male		Female	
	COR (95% CI)	AOR (95% CI)	COR (95% CI)	AOR (95% CI)
Socio-demographics				
Age (years)				
18–29	1 (Reference)	1 (Reference)	1 (Reference)	–
30–49	3.88 (1.52, 9.91)**	2.90 (1.05 7.99)*	1.17 (0.76, 1.79)	
50–69	2.56 (1.02, 6.40)*	2.41 (0.90, 6.46)*	1.14 (0.74, 1.75)	
Education				
Secondary or more	1 (Reference)	–	1 (Reference)	–
Standard 5–8	0.84 (0.31, 2.27)		1.62 (0.89, 2.95)	
Standard 1–4	0.87 (0.34, 2.25)		1.31 (0.77, 2.24)	
None	1.59 (0.22, 11.75)		0.69 (0.35, 1.37)	
Marital status				
Never married	1 (Reference)	–	1 (Reference)	–
Married/cohabiting	1.20 (0.34, 4.27)		1.69 (0.77, 3.70)	
Separated/divorced/widowed	0.59 (0.14, 2.49)		2.00 (0.80, 5.02)	
Employment status				
Nonpaid or unemployed	1 (Reference)	–	1 (Reference)	–
Employed or student	1.23 (0.66, 2.27)		0.71 (0.44, 1.16)	
Residence				
Rural	1 (Reference)	–	1 (Reference)	–
Urban	0.84 (0.25, 2.86)		1.08 (0.63, 1.86)	
Language of interview				
Tumbuka	1 (Reference)	–	1 (Reference)	–
Chewa	0.88 (0.17, 4.57)		1.79 (0.62, 5.18)	
English	1.31 (0.35, 4.85)		2.57 (0.89, 7.41)	
Psychosocial distress				
Alcohol family problem	5.09 (2.51, 10.31)***	5.04 (2.18, 11.65)***	3.11 (1.49, 6.49)**	2.75 (1.34, 5.61)**
Family member attempted suicide	0.98 (0.29, 3.29)	–	2.96 (1.31, 6.66)**	–
Family member died from suicide	0.50 (0.10, 2.44)	–	3.74 (1.32, 10.64)*	3.33 (1.10, 10.07)*
Passive smoking	1.80 (0.75, 4.30)	–	1.38 (0.88, 2.18)	–
Physical factors and risk behaviours				
Systolic blood pressure (< 100 mmHg)	0.90 (0.42, 1.97)	–	2.96 (1.31, 6.68)**	0.96 (0.41, 2.18)
Not taking anti-hypertension medication	0.37 (0.08, 1.71)	–	1.79 (1.01, 3.16)*	1.35 (0.79, 2.30)
Body mass index				
Normal	1 (Reference)	1 (Reference)	1 (Reference)	–
Underweight	0.68 (0.23, 2.01)	0.78 (0.29, 2.11)	1.94 (0.94, 4.01)	
Overweight	1.18 (0.57, 2.46)	1.20 (0.52, 2.70)	0.73 (0.41, 1.28)	
Obesity	0.22 (0.05, 0.87)*	0.15 (0.04, 0.67)*	0.68 (0.35, 1.31)	
Diabetes status				
No	1 (Reference)	–	1 (Reference)	–
Prediabetes	1.20 (0.24, 6.13)		2.46 (0.99, 6.13)	
Diabetes	2.17 (0.43, 10.91)		1.26 (0.37, 4.28)	
Raised total cholesterol	1.96 (0.71, 5.36)	–	0.59 (0.35, 1.01)	–
Heart attack, angina or stroke	0.90 (0.30, 2.68)	–	2.36 (1.28, 4.33)**	2.07 (1.17, 3.66)*
Early smoking initiation (≤ 14 years)	8.60 (2.03, 36.45)**	6.20 (0.75, 51.16)	2.01 (0.21, 19.27)	–
Current smokeless tobacco use	0.17 (0.02, 1.58)	–	1.91 (0.89, 4.14)	–
Current alcohol use	1.50 (0.65, 3.42)	–	2.33 (1.05, 5.14)*	1.48 (0.77, 2.83)
Alcohol dependence	1.76 (0.53, 5.80)	–	0.78 (0.15, 3.99)	–
No active transport	0.31 (0.10, 0.98)*	0.36 (0.11, 1.15)	2.62 (1.58, 4.35)***	2.43 (1.46, 4.06)**
Sedentary behaviour	2.64 (0.96, 7.25)	–	1.11 (0.57, 2.27)	–

*COR* Crude Odds Ratio, *AOR* Adjusted Odds Ratio

***P < 0.001, **P < 0.01, *P < 0.05

## Discussion

In this nationally representative sample of persons aged 18–69 years in Malawi, the lifetime prevalence of suicide attempts (0.8%) was similar to national studies among adults in Nigeria (0.7%) [6], and Bhutan (0.7%) [10], but lower than in South Africa (2.9%) [2]. The prevalence of SB in the past year (7.2% ideation, 3.9% plans, and 0.4% attempt) in this study was higher than in ten lower resourced countries (2.1% ideation, 0.7% plan and 0.4% attempt) [7], but similar to Ethiopia (7.0% ideation%, 4.6% plan and 3.7% attempt) [8]. The considerable proportion of SB in Malawi compares with a prevalence of 12.8% suicidal ideation and 12.9% suicide attempts among adolescents in Malawi [34]. The suicide rate in Malawi in 2016 (7.8 per 100,000) [3] or in 2017 (9.0 per 100,000 [4] was lower than the rate in low-income countries (10.8 per 100,000) [3]. The study found that the lifetime suicide attempt rate in Malawi was similar to Nigeria (in a study conducted from 2002–2003) [6] and Bhutan (in a study conducted in 2014) [10]. Similar socioeconomic and cultural factors [35] may play a role in the similarity of rates in these countries. Malawi is classified as a low-income country, while Nigeria and Bhutan are lower-middle income countries [36].

The study found that psychosocial distress (death of a family member from suicide and alcohol family problems) and female sex were associated with SB in the past year. Major suicide attempt methods were to “hang on a rope”, “overdose of medication” and “poisoning with pesticides”, which are similar to global suicides in low- and middle-income countries [1]. In a retrospective audit of suicides autopsied at a hospital in Malawi showed that the main method of suicide was chemical poisoning by agricultural pesticides [37]. “Knowledge of the most commonly used suicide methods is important to devise prevention strategies that have shown to be effective, such as restriction of access to means of suicide.” [1] Among participants who reported suicidal ideation in the past year in this study, 29.5% got professional care, which is lower than in high-income countries (51.9–60.9%) but higher than low-income countries (13.6–17.8%) [6], and among participants with a suicide attempt, 4.1% got medical treatment which is much lower than in high-income countries (64.7–70.3%), middle-income countries (47.9–49.1%), and low-income countries (14.8–48.1%) [9]. People with mental illness may experience stigma and discrimination, hindering them to access treatment and to prevent suicide [38, 39]. Globally, the most common barriers to not receiving treatment in those with SB include low perceived need in low-income countries (67%) and in high-income countries attitudinal barriers (54%) and low perceived need for treatment (45%) [9]. Access to treatment for SB and/or depression may be improved by expanding primary mental health care [40]. “With severe shortages of mental health professionals in Malawi, integration of mental health into existing primary and community health services is the most feasible way of increasing access to services for people with mental health problems.” [40]

In line with previous findings [7, 8, 10], this survey showed that women and inconsistent with previous research [7, 8, 10] that older age among men had increased odds of SB. However, in a review of studies in Africa, mixed results were found on the female or male preponderance of SB [41]. One possible reason for the higher prevalence of suicidal ideation in men 30 years and older may be related to the higher prevalence of HIV in men 30 years and older in Malawi [42]. Compared with the general population, persons with HIV “show an increased rate of suicidal ideation, suicide attempts, and completed suicide” [43]. However, we could not find significant differences in the prevalence of SB regarding education, marital status, work status, language group, and residence status, while some previous studies [7, 8, 10, 11] found that lower socioeconomic or lower educational status increased the odds of SB.

Having a family history of suicide has been a known risk factor for SB [7, 8, 10], which was confirmed in this study in terms of death of a close family member. In agreement with previous investigations [1, 23–25, 28, 29], this study showed an alcohol family problem and in unadjusted analysis, early smoking initiation among men and current alcohol use among women increased the odds for SB. While some studies [29, 30] found a positive association between overweight or obesity and SB, this study found among men a negative association between obesity and SB. It is possible that in this low-income setting, being overweight is associated with greater well-being and thus protective against SB.

In agreement with previous studies [13, 44], having a history of cardiovascular disease was associated with SB among women. Cardiovascular disease patients may experience a higher rate of disability, which may increase their vulnerability to SB [35, 44]. The study showed in univariate analysis that systolic blood pressure < 100 mmHg and nontaking of antihypertensive medication among women were associated with SB in the past year. In a large previous study in South Korea, systolic blood pressure < 100 mmHg increased the odds of suicidal ideation [19]. The possible relationship between low blood pressure and SB in Africa should be the subject of further research. Contrary to former research [16–18, 20–22], this Malawi survey did not find a significant association between raised total cholesterol, sedentary behaviour, diabetes and SB. However, this study found a significant positive association among women between nonuse of active transportation and SB. This result may confirm an association with one type of sedentary behaviour and SB [20–22].

## Study limitations

Study limitations include the cross-sectional design of the study, which hinders us to draw causative conclusions. In addition, most of the data were assessed by self-report, which may lead to biased responses. Furthermore, certain variables, such as parental psychopathology and mental disorders of the study participants, were not assessed in Malawi STEPS survey and should be included in future research. The variable on household income was not included, because too many values were missing.

## Conclusion

Findings of this nationally representative community survey of persons aged 18 to 69 years in Malawi showed that 0.8% had ever attempted suidide and that almost one in ten had engaged in SB in the past year. Several risk factors of SB for men and/or women were identified, including female sex, older age among men, alcohol family problems (both men and women), death from suicide of family members among women, history of cardiovascular disease and no active transportation among women, which may assist in designing programmes to prevent SB in the Malawian population. Furthermore, the low health care utilization for SB in this study in a low-income country versus high receipt for treatment of SB in high-income countries may call for the integration of mental health into existing primary and community health services to increase access to services for people with mental health problems.

## Supplementary Information

**Additional file 1.** Description of study variables.

## Data Availability

The data for the current study are publicly available at the World Health Organization NCD Microdata Repository (URL: https://extranet.who.int/ncdsmicrodata/index.php/catalog).

## Abbreviations

AUDIT: Alcohol Use Disorder Identification Test
STATA: Statistics and data
STEPS: STEPwise approach to surveillance
SB: Suicidal behaviour
TC: Total cholesterol
