# Supplementary material for: Prevalence and correlates of suicidal behaviour among adults in Malawi: a nationally representative cross-sectional survey in 2017
Source: Int J Ment Health Syst. 2021 Jun 6;15:57. doi: 10.1186/s13033-021-00483-x (PMC8183087; doi:10.1186/s13033-021-00483-x)
Supplement: Supplementary file 1 — Additional file 1. Description of study variables. [file 13033_2021_483_MOESM1_ESM.docx]

Additional file 1: Description of study variables

| **Variables** | **Question** | **Response options (coding scheme)** |
| --- | --- | --- |
|  | **Outcome variables** |  |
| Suicidal ideation (past 12 months) | “During the **past 12 months**, have you seriously **considered** attempting suicide?” | “Yes, No” |
| Help seeking | “Did you seek **professional help** for these thoughts?” | “Yes, No” |
| Suicide plan (past 12 months) | “During the **past 12 months**, have you made **a plan about how** you would attempt suicide?” | “Yes, No” |
| Ever suicide attempt | “Have you **ever attempted suicide**?” | “Yes, No” |
| Suicide attempt (past 12 months) | “During the **past 12 months**, have you **attempted suicide**?” | “Yes, No” |
| Suicide method | “What was the main **method you used** the last time you attempted suicide?” | For example, “poisoning with pesticites (e.g., rat poison, insecticide, weed-killer)” |
| Medical care | “Did you seek **medical care** for this attempt?” | “Yes, No” |
|  | “Were you **admitted to hospital overnight** because of this attempt?” | “Yes, No” |
|  | **Psychosocial distress** |  |
| Alcohol family problems | “During the **past 12 months**, have you had family problems or problems with your partner due to **someone else’s** drinking?” | 1=yes, more than monthly to 4=yes, once or twice and 5=no (1-4=1 and 5=0) |
| Family member attempted suicide | “Has anyone in **your close family** (mother, father, brother, sister or children) ever attempted suicide?” | “Yes, No” |
| Family member died from suicide | “Has anyone in **your close family** (mother, father, brother, sister or children) ever died from suicide?” | “Yes, No” |
| Passive smoking | “During the past 30 days, did someone smoke in your home?” | “Yes, No” |
|  | “During the past 30 days, did someone smoke in closed areas in your workplace (in the building, in a work area or a specific office)?” | “1=Yes, 2=No, 3=Do not work in a closed area” (coded 1=0 and 2-3=0) |
